# Pandemic treaty as an instrument to strengthen global health security: Global health diplomacy at its crux

**DOI:** 10.34172/hpp.42744

**Published:** 2024-03-14

**Authors:** Vijay Kumar Chattu, Rajani Mol, Bawa Singh, K. Srikanth Reddy, Arian Hatefi

**Affiliations:** ^1^Center for Global Health Research, Saveetha Medical College and Hospitals, Saveetha Institute of Medical and Technical Sciences, Saveetha University, Chennai 600077, India; ^2^Department of Occupational Science & Occupational Therapy, Temerty Faculty of Medicine, University of Toronto, Toronto, ON M5G 1V7, Canada; ^3^Department of Community Medicine, Faculty of Medicine, Datta Meghe Institute of Medical Sciences, Wardha-442107, India; ^4^Department of South and Central Asian Studies, School of International Studies, Central University of Punjab, Bathinda, India; ^5^School of Epidemiology and Public Health, University of Ottawa, Ottawa, Canada; ^6^Center for Evidence-Based Diplomacy, Global Health Research and Innovations Canada (GHRIC), Toronto, ON, Canada; ^7^Institute for Global Health Sciences, School of Medicine, University of California San Francisco, San Francisco, CA 94117, Canada

**Keywords:** Pandemic, International Health Regulations (IHR), COVID-19, Infectious diseases, Health security, Diplomacy, Health promotion

## Abstract

**Background:**

The World Health Assembly (WHA), on 1^st^ December 2021, unanimously agreed to launch a global process to draft and negotiate a convention, agreement, or other international instrument under the World Health Organization’s (WHO’s) constitution to strengthen pandemic prevention, preparedness, and response. We aimed to explore the role of global health diplomacy (GHD) in pandemic treaty negotiations by providing deep insight into the ongoing drafting process under the WHO leadership.

**Methods:**

We conducted a narrative review by searching Scopus, Web of Sciences, PubMed, MEDLINE, and Google Scholar search engine using the keywords "Pandemic Treaty," OR "International Health Regulations," OR "International conventions," OR "International treaties" in the context of recent COVID-19 pandemic. Besides, we included articles recommending the need for GHD, leadership and governance mechanisms for this international treaty drafting approved by the WHA.

**Results:**

Amid the COVID-19 pandemic, the concept of GHD bolstered the international system and remained high on the agendas of many national, regional and global platforms. As per Article 19 of the WHO constitution, the Assembly established an intergovernmental negotiating body (INB) to draft and negotiate this convention/ agreement to protect the world from disease outbreaks of pandemic potential. Since GHD has helped to strengthen international cooperation in health systems and address inequities in achieving health-related global targets, there is a great scope for the successful drafting of this pandemic treaty.

**Conclusion:**

The pandemic treaty is a defining moment in global health governance, particularly the pandemic governance reforms. However, the treaty’s purpose will only be served if the equity considerations are optimized, accountability mechanisms are established, and a sense of shared responsibility is embraced. While fulfilling treaty commitments might be complex and challenging, it provides an opportunity to rethink and build resilient systems for pandemic preparedness and response in the future.

## Introduction

 COVID-19 is an unprecedented challenge with detrimental consequences that the world has never witnessed. Pandemics are a major concern to humanity. COVID-19 exposed nation-state public health weaknesses, inequalities, and vulnerabilities and exploited our interconnectedness as the epidemic turned into a pandemic. The current global health governance structure has failed to provide reasonable and equal access to the effective prevention of pandemics, and World Health Organization (WHO) has fallen short of its expectations by its delayed responses, e.g., in 2014 declaring Ebola as a Public Health Emergency of International Concern (PHEIC) at a very late stage and similarly in 2020 declaring COVID-19 as a pandemic after a huge loss of human lives. Preventing the next pandemic requires significant changes to these systems and commitment at the highest level to achieve and sustain them.^1^ COVID-19 taught a lesson that no single government or multilateral agency could address future pandemics alone. Therefore, on March 30, 2021, the Twenty-five heads of government joined the European Council President, Charles Michel, and the WHO Director-General Tedros Adhanom Ghebreyesus to unanimously call for a “new international treaty for pandemic preparedness and response.”^2^ The WHO Framework Convention on Tobacco Control (FCTC) is the only instrument adopted under Article 19 to date that has substantially and rapidly contributed to protecting people from tobacco since its entry into force in 2005 through successful global health diplomacy (GHD).^3^

 The WHO’s International Health Regulations (IHR) were adopted by the World Health Assembly (WHA) in 1969 and last revised in 2005 to “prevent, protect against, control, and respond to the international spread of diseases.^4^ The IHRs have established a stronger legal framework for bolstering global health security (GHS) and international cooperation. It is an international treaty, legally binding on 194 countries across the globe, including all WHO member states.^5^ At the WHA, the 194 members of the WHO adopted on May 31, 2021 the decision to discuss a new international treaty on pandemics at a special session in November 2021.^6^ An international pandemic treaty adopted within the WHO framework would enable countries worldwide to strengthen national, regional, and global capacities and resilience to future pandemics.^7^ The European Council President, reiterated the European Union’s (EU’s) call for an International Treaty to “foster a comprehensive approach to better predict, prevent and respond to pandemics, strengthen global capacity and resilience to ensure fair access to medical solutions, and bolster international alert systems that are sharing cutting-edge medical research”.^8^ The idea behind the proposal for such a treaty is to systematically tackle the gaps exposed by COVID-19.^9^ The question of whether the pandemic treaty would be necessary to deal with a pandemic or whether to revise/improve the current IHR would come up. In this context, the WHA Special Session, which took place from November 29 to December 1, 2021, focused on developing a new WHO-led international instrument on pandemic preparedness and response.^10^ The WHA established an intergovernmental negotiating body (INB) to draft and negotiate this convention/ agreement to protect the world from future infectious disease crises under Article 19 of the WHO constitution.^11^ Subsequently, on February 1, 2023, the WHO published the zero draft of the pandemic treaty emphasizing equity and effectiveness through international cooperation for prevention, preparedness and response against pandemics in the future.^12^ Given this background, this review aims to explore the role of GHD in successful pandemic treaty negotiations. The objectives are (1) to examine the role of GHD in the international pandemic treaty negotiations and (2) to understand how the pandemic treaty helps to strengthen GHS and multilateral health architecture.

## Materials and Methods

 The narrative review approach was employed to answer the research question using the available evidence in the published literature and reports. The narrative method works as a bridge between the existing literature and the current events in a critically acclaimed way.^13,14^ Since the ‘pandemic treaty’ has been a developing concept since 2020, the narrative review seems to be an appropriate methodology to summarize and give a snapshot based on the available published literature from relevant sources. Therefore, we employ this technique to provide critical insight into this important study. Besides, the narrative review is a robust method and powerful tool as it helps explain the current events that may not have been previously observed and are still ongoing.^15^

 The well-debated topic of the ‘pandemic treaty’ and its advantages for global citizens becomes the right topic for conducting cross-disciplinary perspectives among the domains of politics, policymaking, public health, ethics, management, and others. We, therefore, have conducted an extensive literature search for this review by reviewing relevant articles and reports. For this, we included articles recommending the need for GHD, leadership and governance mechanisms for this international treaty approved by the WHA in December 2021. The advantages of this narrative type of literature review are: (1) it paves the way for future literature developments, (2) it converts inductive reasoning into a theoretical building block^16,17^ and (3) it enables cross-disciplinary research and shapes new research avenues,^18^ especially in our current research on the evolution and the need for pandemic treaty. During this pandemic phase, it is very critical and beneficial to provide the narratives of all the perspectives of global stakeholders, regional bodies, and national governments as the pandemic-related events unfold.

 The articles of potential interest were identified by searching popular databases such as Scopus, Web of Sciences, PubMed, MEDLINE, and Google Scholar search engine using the keywords “Pandemic Treaty,” OR “International Health Regulations,” OR “International conventions,” OR “International treaties” in the context of recent COVID-19 pandemic. The search was then refined by identifying articles that discussed the need and role of a new international instrument for preventing future disease epidemics/ health threats. Though the emphasis was given to published articles, some authentic websites (such as WHO) and other technical reports were also included. To ensure the quality, two investigators (VKC and BS) independently assessed the articles by excluding the duplicates from the final search. This review provides deep insight into the ongoing process of pandemic treaty drafting, GHD and global leadership issues. The paper explores the need and role of the pandemic treaty, the need for GHD and strong global leadership for a post-pandemic world through the standpoints of equity and bioethics by dynamically exploring contemporary research. The main findings from the literature search are described below in the results section.

## Results

###  Why a pandemic treaty now?

 The WHA has the right to establish conventions or agreements on any topic under WHO’s jurisdiction, according to Article 19 of the WHO Constitution. If a new binding instrument were negotiated, it should help to address some of those weaknesses and contribute to establishing a stronger international health framework, with WHO as the governing authority for global health not only *de facto* but *de jure.*^7^ Such an instrument should be based on principles of collective solidarity, anchored in the principles of equity, fairness, inclusiveness, and transparency.^6^ Therefore, it is very critical that the countries adhere to such a treaty in their self-interest. Moreover, the behavior is not likely to change without a strong value proposition for each country. The proposed pandemic treaty should be the right place to address some of the shortcomings of the IHR, such as concerning independent verifications, inspections, monitoring, enforcement, and compliance, along with ambiguities about travel restrictions, which would be one important cluster of issues to cover.^4^

 The new pandemic treaty calls for a One Health approach that involves the integrated control of human and animal diseases, taking into account the occurrence of interspecies transmission.^19^ The principal aim of a pandemic treaty should be to go beyond, rather than merely facilitate, the application of IHR. The pandemic treaty could contain a preambular reference to Article 57 of IHR to underline links and complementary elements between the two.^4^ The treaty could also contain a substantive article or an optional protocol, giving the treaty force to relevant provisions of the regulations. Measures should also go beyond the current scope of IHR to cover the approval, production and supply of- and access to relevant medical equipment, vaccines, diagnostics, and medicines; the capacity of healthcare sectors in pandemics, including international support when critically needed; cooperation on research and technology sharing; and the intersection of pathogens.^20^

 The proposed pandemic treaty should complement, not replace (or repeat measures already in) the IHR. The treaty should aim at not only the shortcomings of IHR revealed during the COVID-19 response but also measures much beyond the current scope of the IHR.^4^ The INB formed to negotiate the treaty held its first meeting before 1 March 2022 to agree on timelines and its second meeting by 1 August 2022 to discuss progress on a working draft.^21^ The INB, which was tasked to draft and negotiate this international instrument on pandemic prevention, preparedness and response, confirmed that most of the WHO member states favored a legally binding instrument. However, several member states expressed support for a legally binding instrument to be adopted under Article 19 (which gives WHO a wider scope to address). In contrast, some member states, including the United States, want to keep Article 21 (refer Box 1), and Russia preferred Article 23.^22^

**Box 1. Differences between Article 19 and Article 21 of WHO’s Constitution**

**Article 19**

1. The most comprehensive provision of the treaty
2. Allows the WHA to adopt agreements and treaties on any matter within WHO’s competence
3. It virtually covers any issue related to health
4. Examples include the FCTC
5. Provides scope for wider issues such as zoonotic diseases, AMR and Climate change as health threat
6. It requires a 2/3^rd^ vote of WHA, which can be challenging if powerful states oppose it
7. The treaty has a separate and independent secretariat different from the hosting organization

**Article 21**

1. Limits binding agreements to just a few topics
2. Allows only five areas - sanitation and quarantine, disease nomenclature, diagnostic procedures, standards for safety and efficacy and advertising and labeling of drugs and medical products
3. It has a limited scope compared to Article 19
4. Examples include International Health Regulations
5. Regulations enter into force for all member states at the same time as per the deadline set by WHO DG
6. It requires a simple majority vote for adoption
7. Regulations are embedded in WHO’s governance, thereby having WHO as its secretariat

World leaders’ willingness to act provides a historic window of opportunity for GHD to create a new rulebook for collective action.^20^ The treaty aims to instill a political commitment to health security compliance among states through the language of global collaboration, cooperation, and solidarity to mitigate the effects of future pandemics.^23^The key statements by various experts on pandemic treaty are highlighted (Box 2).

**Box 2. Key statements made by the health diplomats in the context of the pandemic treaty**

“*The pandemic is a crisis of solidarity that has been exposed and exacerbated by fundamental weaknesses in the global health architecture: complex and fragmented governance; inadequate financing; and insufficient systems and tools. The only way we can solve them is with a binding treaty or agreement between nations.” Such an accord “could set out high-level, agreed principles to strengthen solidarity, equity, [and] one health for all”*- Tedros Adhanom Ghebreyesus, WHO Director-General.

“*There is a lot of traction on trying to embark on having a binding instrument that brings people together who have been separated in a way. We’ve seen a very strong decoupling in global health over the last two years, so there is a need to integrate many of the initiatives.*”- Ilona Kickbusch, Global Health Centre (Geneva, Switzerland)

“*I think one thing seems quite clear now, there's enough support to begin negotiations over a legally binding agreement, like the [2003] Framework Convention on Tobacco Control, that's been pushed very hard by Europe and the friends of the treaty”-* Lawrence Gostin, Georgetown University (Washington, DC, USA)

“*If there is authorization to embark on a treaty, as they did with the Framework Convention on Tobacco Control, the issues that WHO would want to ensure would be more equitable distribution of goods either during a pandemic or during a preparedness stage and I think that's a worthy goal as that's not covered in the IHR. The IHR does not talk about equity of different goods, and it's been difficult.*”- David Heymann, London School of Hygiene & Tropical Medicine (London, UK)

“*Though IHR stipulates that governments should not impose travel bans or close borders, governments have closed frontiers and grounded flights in efforts to contain the nascent omicron variant. We have these treaties, and we can have a new one, but unless you change the incentives for governments to comply with such obligations, the story will be the same”-* Andrés Constantin, O’Neill Institute for National and Global Health Law at Georgetown University

###  The rising global infectious and climate change threats 

####  Infectious diseases

 Disease outbreaks are a never-ending and intensifying challenge that can emerge or re-emerge in unpredictable regions and times. The modeling studies suggest a high probability of occurrence of disease outbreaks with pandemic potential in the future.^24^ The continued emergence or re-emergence of the viral disease has a detrimental impact on health, economy, social security, and stability worldwide.^25^ COVID-19 affected the entire health system through its direct effect as a communicable disease, as well as its ability to alter the overall mortality and burden of disease through its impact on non-communicable diseases (NCDs).^26^ In the context of GHS and multilateral health architecture, a pandemic treaty might help the prevention, preparedness and response to future pandemics. Therefore, a pandemic treaty (an international agreement) with commitments from countries for collective action for future pandemic prevention is required to strengthen the global health framework. Indeed, the treaty discussions provide an opportunity and an imperative to rethink the paradigm of GHS that has shaped the current international response to the COVID-19 pandemic.^27^ The COVID-19 pandemic has shone a harsh light on the world’s economic and social order, exacerbating inequities that have devastated those most vulnerable and calling into question our ability to cooperate and collaborate through global solidarity even when confronted with a common crisis. Throughout the pandemic, fragmented global and national responses floundered because of gaps in technical skill or capacities and failures in governance and leadership to apply systems-thinking, human rights-based, and health-in-all-policies approaches. Therefore, sustained political leadership and effective governance were key factors in the COVID-19 response and will continue to be important in future preparedness efforts. Good governance requires that health decision-making processes and institutions at the international and national levels are accountable, transparent, equitable, inclusive, participatory, and consistent with the rule of law.

###  Critical role of global health diplomacy

 The COVID-19 is a catastrophic pandemic that exposed the failure of international cooperation. GHD, as a catalyst for global cooperation and collaboration in this current predicament, bolstered the international system and remains high on the agendas of many nations’ regional and global platforms. Health diplomacy is a multi-actor, multi-level interaction that shapes and influences the global health environment. Nation-State remains central to health diplomacy, especially in cooperation with various stakeholders- state, non-state and multilateral actors, and non–governmental organizations.^28^ Besides, the private sector could use greater attention going forward, particularly around R & D and supply chains. More precisely, Fauci defines health diplomacy as “winning the hearts and minds of people in poor countries by exporting medical care, expertise and personnel to help those who need it most.^29^ Schrecker states that GHD combines the art of diplomacy with the science of public health; it balances concrete national interest with the abstract collective concern of the larger international community’s health; it reduces health inequalities; it secures human rights; and it recognizes that effective international health interventions are ethical and sensitive to historical, political, social-economic and cultural differences.^30^ Fidler writes that GHD aims to capture “policy-shaping and negotiate responses to health issues or use health principles or framework in policy shaping and negotiations techniques, to achieve other political, economic, social goals. This definition acknowledges the dual nature of the relationship between health and foreign policy and the importance of negotiation in achieving objectives within each policy sphere.^31^

 GHD remained an esoteric tool for many years. COVID-19 has exposed the global health system’s long-term fragilities, inequalities, injustices, and fragile nature. The outbreak of COVID-19 distorted human life and crippled and stagnated the economy and global supply chain system. The emergence of COVID-19 has accelerated the rise of health on the foreign policy agenda. During the COVID-19 pandemic, many countries and regions worldwide have adopted health diplomacy as a diplomatic priority and strategic foreign policy tool. As a result of the COVID-19 pandemic, various nations reinforced their GHD through vaccine diplomacy, medical diplomacy, mask diplomacy, and humanitarian assistance. And nations followed predominantly bilaterally-oriented health diplomacy while donating pharmaceutical products, personal protective equipment (PPE) including masks, gloves, disposable gowns, face shields, testing kits, etc.

 The pandemic could be an opportunity for the scope of GHD and four key areas of impact: international cooperation, health security, the health system, and economy and trade.^32^ Lack of global cooperation and coordination obstructed a successful and integrated global response to COVID-19 worldwide. In this context, GHD brings all global stakeholders together on a common platform to combat the pandemic; nations, including state and non-state entities, should unite to guarantee a safer world. GHD can serve as a bridge for international collaboration in addressing public health crises and improving health systems by stressing universal health coverage (UHC) to achieve sustainable and equitable development and rebuilding multilateral organizations.^33^ Successful GHD will assist in achieving the disease-specific national goals and the attainment of health-related sustainable development goals (SDGs) and UHC at the global level^34^. As emphasized by Hoffman, effective global governance is not possible when countries cannot depend on each other to comply with international agreements.^35^ WHO FCTC^3^ and the Port of Spain Declaration on NCDs^36^ are excellent examples of successful negotiations of GHD.

 Health has become a major national and global security concern in a globalized world. The world’s interconnectedness has become increasingly vulnerable to infectious diseases, seriously threatening GHS. Health diplomacy holds great promise to address the needs of GHS through its binding or nonbinding instruments enforced by global governance institutions.^37^ The WHO stated that ‘functioning health systems are the bedrock of health security’.^38^ Due to the growing interdependence among countries, most global policies for health are international, which aim to safeguard the health and well-being of people. For instance, the Global Health Security Agenda (GHSA) is an excellent example of successful health diplomacy.

 The pandemic exposed the weakness and vulnerability of health systems in developed and developing countries. The global shortage of medical equipment and a well-equipped and sufficient number of healthcare workers posed major barriers to the health system. During the pandemic, some developed countries with the best health systems are crumbling and struggling to contain the virus, while some developing countries are doing much better. More than ever, governments must be politically committed to achieving “health for all” and promoting the one health approach to reduce the risk of future infectious diseases and improve the global response to pandemics.^32^ Therefore, to mitigate the pandemic’s worst impact and implement these key policies, there is a great need for GHD. For successful GHD, the health system requires collective action by experts, leaders and policymakers from different regions and across different fields of expertise, and it improves the health system.

 COVID-19 has severely affected the global economy and supply chain by creating a negative economic impact due to reduced productivity, a rise in unemployment, trade disruption, business closure, disruption in the service and manufacturing industry, etc. According to Antonio Guterres, the secretary-general of the UN, the pandemic has “exposed long-term fragilities, inequalities, and injustices as the world experienced its worst recession in eight decades with increasing severe poverty and the danger of famine, the social and economic effect of a pandemic is huge and growing”.^39^ India and South Africa have developed a proposal to the WTO to waive some of the requirements under the Agreement on Trade-Related Aspects of Intellectual Property Rights (TRIPS) to support the manufacture of medical products to aid the COVID-19 pandemic response.^40^ However, during the WTO TRIPS Council meeting on October 16, 2020, this proposal was rejected by nine WTO members, including the European Union, although 100 countries showed support for the proposal.^41^ As part of the Doha Declaration, the TRIPS Agreement and Public Health have stated key flexibilities to countries in Article 31.^42^

 Furthermore, clause 5(c) of the TRIPS Agreement stated that “public health crises, including those relating to HIV/AIDS, tuberculosis, malaria and other epidemics,” can constitute “a national emergency or other circumstances of extreme urgency.”^43^ The clause provides countries with some flexibility in managing the patents for pharmaceuticals (public goods), especially in situations of “national emergencies” and “other circumstances of extreme urgency”.^44^ To succeed and ensure access, India and South Africa must get more engaged in GHD with all the involved global stakeholders to get strong support for their joint proposal.^45^ Although international trade cooperation has suffered geopolitical rivalry and shifts, governments can strengthen the nexus between public health and trade policies through GHD to fight the pandemic.^33^ GHD can strengthen international cooperation and health systems, improve the global economy trade, and address the inequities to achieve health-related global targets. Therefore, GHD can deal with several complex issues in the multipolar world that strongly link geo-socio-economic and political determinants and pave the way for health, development, security, and peace.^32^ Research by various authors have stressed that GHD has the potential to address global vaccine inequities,^46,47^ access to medicines^48^ issues of trade and health^49^ and strengthen health systems.^50^ COVID-19 may be a “wake-up” call for global leaders to intensify cooperation on epidemic preparedness and provide the necessary financing for international collective action.^51^ The epidemic and current pandemic highlighted the necessity of GHD and international collaboration as crucial tools to cope with an emerging challenge.

## Discussion

###  Global strategies to address global health security

 International organizations and governments have formulated various strategies to improve and safeguard GHS. The most fundamental reforms of the international system to strengthen GHS are IHRs, GHSA, the FCTC, SDGs and Global Health Partnerships, and the Independent Panel for Pandemic Preparedness and Response (IPPPR). Under IHR, states are required to develop, strengthen and maintain core public health capacities for surveillance and response and to notify, assess, report, and respond to PHEICs.^7^

 GHSA is a USA-led diplomatic initiative formally launched on 13 February 2014, and seventy countries have signed onto the GHSA framework. The GHSA aims to accelerate toward a world safe and secure from infectious disease threats and promotes GHS as an international priority.^52^ It aims to spur progress towards the implementation of the WHO’s IHRs, the World Organization for Animal Health (OIE) Performance of Veterinary Services (PVS) pathway, and other relevant GHS frameworks.^53^ “GHSA 2024” is the strategic framework of all member countries launched in 2018. GHSA 2024 positions member countries to develop the leadership, technical knowledge, and collaborative foundation to sustain health security in the long term.^54^

 In September 2015, the United Nations General Assembly adopted 17 SDGs with the intent to be achieved by the year 2030.^55^ SDG 3 deals with Health and well-being and placed a central position in the SDGs. The goals include: to reduce global maternal mortality, to end preventable deaths of new-borns and children, prevention of harmful use of alcohol, death, and illness from hazardous chemicals and air, water, and soil pollution. Besides, they specifically address reducing the burden of communicable diseases and NCDs, ensuring universal access to sexual and reproductive health care services, achieving UHC, strengthening mental health and well-being, strengthening WHO’s FCTC in all countries and encouraging research and development of vaccines and medicines for the communicable and NCDs.^56^

 WHO’s FCTC is the first treaty negotiation under the auspices of 168 WHO member states and the United Nations. FCTC is one of the widely recognized legally binding treaties and a milestone in the history of GHS. It was adopted by the WHA in May 2003 and entered into force on 27 February 2005. The FCTC was developed in response to the globalization of the tobacco epidemic.^3^ The treaty addresses a ban on tobacco advertising, sponsorship, promotion, and secondhand smoke in all public places, reducing the smuggling of tobacco products, disclosure and regulation of ingredients in tobacco products, sale of tobacco products by or to minors, treatment for tobacco addiction, research and exchange of information among countries and promoting public awareness.^3^ The Framework Convention represents a new approach to international health cooperation, with a legal framework to shape the future of health for all people.^57^ The WHO’s FCTC offers a model for addressing the negative effects of globalization on health.^58^

 In May 2020, WHO initiated an independent panel for pandemic preparedness and response to understand the international health response to the pandemic. On May 12, 2021, the panel presented its findings and recommendations to curb the COVID-19 pandemic and ensure that any future infectious disease outbreak does not become another catastrophic pandemic.^59^ The independent panel’s recommendations have elevated leadership to prepare for and respond to global health threats to the highest levels to ensure just, accountable and multisectoral action. Focus and strengthen the independence, authority, and financing of the WHO. Invest in preparedness now to create fully functional capacities at the national, regional, and global levels, establish a new international system for surveillance, validation, and alert, establish a pre-negotiated platform for tools and supplies, raise new international financing for the global public goods of pandemic preparedness and response, Countries to establish the highest-level national coordination for pandemic preparedness and response.^60^ The Panel believes that system-level change is needed to overcome the manifest failure of the international system to prevent, contain, and mitigate the impact of a pandemic. Pandemic preparedness and response have to function at national, regional, and global levels across different sectors of social and economic life, including government, business, and community.^61^ To prepare the world for the future so that the next disease outbreak does not become a pandemic, the panel calls for a series of crucial reforms that will address gaps in high-level coordinated leadership globally and nationally, funding, access to what must become global goods, and WHO’s independence, focus, and authority.^62^

###  Strong leadership for implementation of the pandemic treaty 

 A more nuanced point is contained in the idea that “epidemics are inevitable while pandemics are optional.” Both prevention and mitigation seem squarely within scope. However, one possible example of failure in the global governance structure is the possibility that there was a large lead time between concern raised in Wuhan and countries like Italy, the United States, or those in MENA failing to put up necessary safeguards- this seems to be where the IHR failed, and where a stronger instrument might succeed. The IHRs are largely built on the assumption that disease outbreaks cannot be prevented; they can only be contained and extinguished. A global pandemic treaty should focus on reducing the risk of pathogens transmission from animals to humans and aim for deep prevention of future pandemics.^63^ Deep prevention focuses on preventing the outbreak of the disease from occurring rather than focusing on a local, national, or international spread.^64^

 The pandemic treaty needs to meet at least two recommendations. More than 30 countries and every member of the European Union endorse the international pandemic treaty, which is also backed by the African Union, Asian, and South American governments.^65^ Leaders from the United States, China, and Russia did not sign the treaty or framework on pandemic preparedness and response.^66^ So yet, only a few countries have taken this step. First, countries from the Global North and South should agree and sign an international treaty for pandemic preparedness and response. Pandemic preparedness needs global leadership for a global health system fit for this millennium.^9^ The zero draft of the pandemic treaty is being consulted for inputs from the member states, so broader stakeholder engagement is warranted. However, inadequate stakeholder engagement and participation appeared to be a limitation in a few countries, regardless of the global south or north. It is argued that the “*governments wanted to hear what they wanted to hear rather than what needed to be said*” by the stakeholders in member states.^67^

 In the ongoing pandemic treaty negotiations, while most WHO member states favored a legally binding instrument, there were differences among the member states on the adoption procedures and corresponding articles of the WHO constitution. The differences appeared visible, as some member states favor Article 19, which gives WHO a wider scope to address health issues, while others, including the United States, favor Article 21, which limits binding agreements to just a few topics. However, only Russia preferred non-legally binding recommendations under Article 23 of the constitution. As the treaty is being drafted and member states have already expressed their preferences on how they want to see a treaty; GHD has a more vital role than ever in building consensus among the leaders of member states.

 Secondly, the Global South is considered a poorer and more unequal country badly affected by COVID-19, with rudimentary social protection systems and often less advanced economies and institutions. Countries in the Global South have been affected differently by the pandemic compared to the Global North. As vaccination campaigns start to roll out, prioritizing countries in the Global North, it is possible that the pandemic will last longer and have deeper impacts in the Global South as countries wait for access to the vaccine.^68^ On 23^rd^ August, it was reported that 32.5% of the world population had received at least one dose of a COVID-19 vaccine, with 24.5% fully vaccinated. However, only 1.4 % of people in low-income countries (LICs) have received at least one dose. Many of these LICs could be considered part of the Global South.^69^ Both the Global North and Global South should have equal access and rapid access to vaccines, thereby addressing the interests of the Global South. Therefore, a new pandemic treaty is essential, as it would commit advanced economics to be more cooperative with less fortunate nations. According to the resolution on December 1, 2021, the INB conducted its first meeting on March 1, 2022 (to agree on working methods and timetables) and its second on August 1, 2022 (to discuss progress on a working draft). It also held public hearings to inform its deliberations, present a progress report to the 76th WHA in 2023, and present its findings to the 77th WHA in 2024.^11^ There is still a long way ahead as we adopt the treaty with equity considerations.^70^

## Conclusion

 The COVID-19 pandemic has shown vulnerabilities in national, regional, and global preparedness and response systems and the potential to improve the global health architecture particulary with regard to health security. GHS. Further, it has demonstrated the necessity for global collective and coordinated action to defend public health and ensure that all countries’ needs, particularly those of developing and least developed, are advanced and safeguarded. The pandemic treaty that is being negotiated should address some of the systematic failures of the present system and enable nations to equitably share their expertise, equipment, and knowledge as we prepare for the next pandemic. And in doing so, specific and measurable equity metrics are found essential.

 The pandemic treaty shall make countries more accountable to one another through their agreed commitments and connecting them in ways that strengthen GHS. Many global health scholars and practitioners sought robust accountability mechanisms in the pandemic treaty, as COVID-19 and other PHEICs presented accountability limitations in the IHRs. We can succeed only if nations work together with shared responsibility by focusing on shared interests in global health. The pandemic treaty provides an opportunity to enhance accountability, solidarity and commitment to collective action for GHS. The pandemic treaty aimed to strengthen GHS as an international priority and ensure that security frameworks are implemented by governments, emphasizing the importance of GHD and its emerging role. Finally, the paper concludes with the words of WHO chief Tedros Adhanom Ghebreyesus: “The time has come to act. The world cannot afford to wait until the pandemic is over to start planning for the next one. We must leave a legacy for our children: a safer world for all”.

## Acknowledgements

 The researchers thank “Global Health Research and Innovations Canada (GHRIC)” for providing administrative and coordination support for this project.

## Competing Interests

 Vijay Kumar Chattu is one of the editorial board members of *Health Promotion Perspectives*. He is also the Founder and CEO of Global Health Research and Innovations Canada (GHRIC), Toronto. Other authors declare no competing interests.

## Ethical Approval

 Not applicable.

## References

[1] Global Preparedness Monitoring Board (GPMB). Statement Ahead of the 74th World Health Assembly. Available from: https://www.gpmb.org/news/news/item/21-05-2021-statement-ahead-of-the-74th-world-health-assembly. Accessed May 21, 2023.

[2] World Health Organization (WHO). WHO Director general’s Remarks at the Press Conference with President of the European Council to Discuss the Proposal for an International Pandemic Treaty. WHO; 2021. Available from: https://www.who.int/director-general/speeches/detail/who-director-general-s-remarks-at-the-press-conference-with-president-of-the-european-council-to-discuss-the-proposal-for-an-international-pandemic-treaty. Accessed March 30, 2023.

[3] WHO Framework Convention on Tobacco Control (WHO FCTC). WHO Framework Convention on Tobacco Control Overview. WHO FCTC; 2003. Available from: https://www.who.int/tobacco/framework/WHO_FCTC_english.pdf. Accessed March 30, 2023.

[4] Nikogosian H, Kickbusch I. How Would a Pandemic Treaty Relate with the Existing IHR (2005)? BMJ Blogs; 2021. Available from: https://blogs.bmj.com/bmj/2021/05/23/how-would-a-pandemic-treaty-relate-with-the-existing-ihr-2005/. Accessed May 23, 2023.

[5] World Health Organization (WHO). International Health Regulations (2005). Geneva: WHO; 2008. Available from: https://www.who.int/publications/i/item/9789241580410. Accessed May 16, 2023.

[6] European Council. An International Agreement on Pandemic Prevention and Preparedness. European Council; 2021. Available from: https://www.consilium.europa.eu/en/policies/coronavirus/pandemic-treaty/. Accessed May 20, 2023.

[7] Velásquez G, Syam N. A New WHO International Treaty on Pandemic Preparedness and Response: Can It Address the Needs of the Global South? South Centre Policy Brief, 93. 2021. Available from: https://www.southcentre.int/wp-content/uploads/2021/05/PB-93-A-New-WHO-International-Treaty-on-Pandemic-Preparedness-and-Response-REV-2.pdf. Accessed May 24, 2023.

[8] Cullinan K. Pandemic Treaty Discussion Deferred with Appeals for High-Level Political Commitment to Fix WHO. Health Policy Watch; 2021. Available from: https://healthpolicy-watch.news/86660-2/. Accessed May 25, 2023.

[9] World Health Organization (WHO). COVID-19 Shows Why United Action is Needed for a More Robust International Health Architecture. WHO; 2021. Available at: https://www.who.int/news-room/commentaries/detail/op-ed---covid-19-shows-why-united-action-is-needed-for-more-robust-international-health-architecture. Accessed May 30, 2023.

[10] Zarocostas J (2021). Countries prepare for pandemic treaty decision. Lancet.

[11] World Health Organization (WHO). World Health Assembly Agrees to Launch Process to Develop Historic Global Accord on Pandemic Prevention, Preparedness and Response. WHO; 2021. Available from: https://www.who.int/news/item/01-12-2021-world-health-assembly-agrees-to-launch-process-to-develop-historic-global-accord-on-pandemic-prevention-preparedness-and-response. Accessed April 20, 2023.

[12] World Health Organization (WHO). Zero Draft of the WHO CA + for the Consideration of the Intergovernmental Negotiating Body at its Fourth Meeting. WHO; 2023. Available from: https://apps.who.int/gb/inb/pdf_files/inb4/A_INB4_3-en.pdf. Accessed June 6, 2023.

[13] Webster J, Watson RT (2002). Analyzing the past to prepare for the future: writing a literature review. MIS Q.

[14] Forsgren M (2018). The development of international business: a narrative of theory and practice. J Int Bus Stud.

[15] Pentland BT (1999). Building process theory with narrative: from description to explanation. Acad Manage Rev.

[16] Ar AY, Abbas A (2021). Public-private ICT-based collaboration initiative during the COVID-19 pandemic: the case of Ehsaas Emergency Cash Program in Pakistan. Braz Arch Biol Technol.

[17] Baumeister RF, Leary MR (1997). Writing narrative literature reviews. Rev Gen Psychol.

[18] Ferrari R (2015). Writing narrative style literature reviews. Medical Writing.

[19] Zaręba S. Towards a Global Pandemic Treaty. Polish Institute of International Affairs (PISM); 2021. Available from: https://pism.pl/publications/Towards_a_Global_Pandemic_Treaty. Accessed April 30, 2023.

[20] Nikogosian H, Kickbusch I. A Pandemic Treaty: Where Are We Now That the Leaders Have Spoken? BMJ Blogs; 2021. Available from: https://blogs.bmj.com/bmj/2021/04/26/a-pandemic-treaty-where-are-we-now-that-the-leaders-have-spoken/. Accessed April 26,2021.

[21] Taylor L (2021). World Health Organization to begin negotiating international pandemic treaty. BMJ.

[22] Ravelo JL. Majority of WHO Member States Want Legally Binding Pandemic Instrument. Devex News; 2022. Available from: https://www.devex.com/news/majority-of-who-member-states-want-legally-binding-pandemic-instrument-103669. Accessed June 6, 2023.

[23] Wenham C. A Global Pandemic Treaty Won’t Work Until Leaders Realize the Benefits of Solidarity. The Guardian; 2021. Available from: https://www.theguardian.com/commentisfree/2021/apr/01/global-pandemic-treaty-covid-disease-control. Accessed April 1, 2023.

[24] Marani M, Katul GG, Pan WK, Parolari AJ (2021). Intensity and frequency of extreme novel epidemics. Proc Natl Acad Sci U S A.

[25] Abebe GM (2020). Emerging and re-emerging viral diseases: the case of coronavirus disease-19 (COVID-19). Int J Virol AIDS.

[26] Azarpazhooh MR, Morovatdar N, Avan A, Phan TG, Divani A, Yassi N (2020). COVID-19 pandemic and burden of non-communicable diseases: an ecological study on data of 185 countries. J Stroke Cerebrovasc Dis.

[27] Fukuda-Parr S, Buss P, Ely Yamin A (2021). Pandemic treaty needs to start with rethinking the paradigm of global health security. BMJ Glob Health.

[28] Novotny TE, Kickbusch I, Told M. 21st Century Global Health Diplomacy. Vol 3. World Scientific; 2013.

[29] Fauci AS (2007). Lasker Public Service Award. The expanding global health agenda: a welcome development. Nat Med.

[30] Schrecker T, Labonté R, De Vogli R (2008). Globalisation and health: the need for a global vision. Lancet.

[31] Lee K, Kamradt-Scott A. Global health diplomacy: a conceptual review. Glob Health Gov 2011;5(1). Available from: https://summit.sfu.ca/item/10888. Accessed April 28, 2023.

[32] Taghizade S, Chattu VK, Jaafaripooyan E, Kevany S (2021). COVID-19 pandemic as an excellent opportunity for global health diplomacy. Front Public Health.

[33] Javed S, Chattu VK (2020). Strengthening the COVID-19 pandemic response, global leadership, and international cooperation through global health diplomacy. Health Promot Perspect.

[34] United Nations. The Sustainable Development Goals Report 2019. United Nations; 2019. Available from: https://unstats.un.org/sdgs/report/2019/The-Sustainable-Development-Goals-Report-2019.pdf. Accessed May 14, 2023.

[35] Hoffman SJ (2010). The evolution, etiology and eventualities of the global health security regime. Health Policy Plan.

[36] Chattu VK, Knight AW (2019). Port of Spain Summit Declaration as a successful outcome of global health diplomacy in the Caribbean region: a systematic review. Health Promot Perspect.

[37] Chattu VK (2017). The rise of global health diplomacy: an interdisciplinary concept linking health and international relations. Indian J Public Health.

[38] World Health Organization (WHO). World Health Day 2007: International Health Security: “Invest in Health, Build a Safer Future”. WHO; 2007. Available from: https://www.who.int/news/item/29-03-2007-world-health-day-2007-international-health-security. Accessed March 19, 2023.

[39] Mol R, Singh B, Chattu VK, Kaur J, Singh B (2022). India’s health diplomacy as a soft power tool towards Africa: humanitarian and geopolitical analysis. J Asian Afr Stud.

[40] World Trade Organization (WTO). TRIPS and Public Health. WTO; 2020. Available from: https://www.wto.org/english/tratop_e/trips_e/pharmpatent_e.htm. Accessed April 24, 2023.

[41] de Menezes HZ. The TRIPS Waiver Proposal: An Urgent Measure to Expand Access to the COVID-19 Vaccines. South Centre Research Paper, 129. 2021.Available from: https://www.southcentre.int/wp-content/uploads/2021/03/RP-129.pdf. Accessed April 24, 2023.

[42] World Trade Organization (WTO). Waiver From Certain Provisions of the TRIPS Agreement for the Prevention, Containment, and Treatment of COVID-19 (IP/C/W/669). WTO; 2020. Available from: https://docs.wto.org/dol2fe/Pages/SS/directdoc.aspx?filename = q:/IP/C/W669.pdf&Open = True. Accessed June 2, 2023.

[43] Wong H (2020). The case for compulsory licensing during COVID-19. J Glob Health.

[44] United Nations Development Programme (UNDP). Using TRIPS Flexibilities to Improve Access to HIV Treatment. UNDP; 2015. Available from: https://www.undp.org/content/undp/en/home/librarypage/hiv-aids/using_trips_flexibilitiestoimproveaccesstohivtreatment.html. Accessed March 25, 2023.

[45] Chattu VK, Singh B, Kaur J, Jakovljevic M (2021). COVID-19 vaccine, TRIPS, and global health diplomacy: India’s role at the WTO platform. Biomed Res Int.

[46] Singh B, Kaur J, Chattu VK (2022). Global vaccine inequities and multilateralism amid COVID-19: reconnaissance of global health diplomacy as a panacea?. Health Promot Perspect.

[47] Singh B, Chattu VK (2021). Prioritizing ‘equity’ in COVID-19 vaccine distribution through global health diplomacy. Health Promot Perspect.

[48] Chattu VK, Singh B, Pattanshetty S, Reddy S (2023). Access to medicines through global health diplomacy. Health Promot Perspect.

[49] Chattu VK, Pooransingh S, Allahverdipour H (2021). Global health diplomacy at the intersection of trade and health in the COVID-19 era. Health Promot Perspect.

[50] Chattu VK, Pooransingh S (2020). Strengthening African health systems through global health diplomacy. Health Promot Perspect.

[51] Pak A, Adegboye OA, Adekunle AI, Rahman KM, McBryde ES, Eisen DP (2020). Economic consequences of the COVID-19 outbreak: the need for epidemic preparedness. Front Public Health.

[52] Centers for Disease Control and Prevention (CDC). Global Health Security Agenda: Action Packages. CDC; 2016. Available from: https://www.cdc.gov/globalhealth/security/actionpackages/default.htm. Accessed January 21, 2023.

[53] Chattu VK, Kevany S (2019). The need for health diplomacy in health security operations. Health Promot Perspect.

[54] Global Health Security Agenda (GHSA). 2024 Framework. GHSA; 2018. Available from: https://ghsagenda.org/wp-content/uploads/2020/06/ghsa2024-framework.pdf. Accessed March 25, 2023.

[55] United Nations. Transforming Our World: The 2030 Agenda for Sustainable Development. United Nations; 2016. Available from: https://sustainabledevelopment.un.org/post2015/transformingourworld/publication. Accessed March 25, 2023.

[56] United Nations Development Programme (UNDP). What Are the Sustainable Development Goals? UNDP; 2015. Available from: https://www.undp.org/sustainable-development-goals?utm_source = EN&utm_medium = GSR&utm_content = US_UNDP_PaidSearch_Brand_English&utm_campaign = CENTRAL&c_src = CENTRAL&c_src2 = GSR&gclid = Cj0KCQjw18WKBhCUARIsAFiW7JyujAV9DaYEwTg-vq0IGUI0zl-rfLkAYrkFn2MqxUSYXmaXcavMvusaAs7aEALw_wcB. Accessed April 25, 2023.

[57] Nikogosian H (2010). WHO Framework Convention on Tobacco Control: a key milestone. Bull World Health Organ.

[58] Nikogosian H, Luiza da Costa e Silva V (2015). WHO’s first global health treaty: 10 years in force. Bull World Health Organ.

[59] The Independent Panel for Pandemic Preparedness & Responses (IPPPR). Main Report & Accompanying Work. IPPPR; 2021. Available from: https://theindependentpanel.org/mainreport. Accessed April 25, 2023.

[60] Mattehret. UN Report Pushes Global Government to” Prevent Future Pandemics”. May 13, 2021. Available from: https://canadianpatriot.org/2021/05/13/un-report-pushes-global-government-to-prevent-future-pandemics/. Accessed May 13, 2023.

[61] Center for Vaccine Ethics and Policy (CVEP). Vaccines and Global Health: The Week in Review. CVEP; 2021. Available from: https://centerforvaccineethicsandpolicy.files.wordpress.com/2021/05/vaccines-and-global-health_the-week-in-review_15-may-2021.pdf. Accessed May 13, 2023.

[62] Sirleaf EJ, Clark H (2021). Report of the Independent Panel for Pandemic Preparedness and Response: making COVID-19 the last pandemic. Lancet.

[63] Vinuales J, Moon S, Le Moli G, Burci GL (2021). A global pandemic treaty should aim for deep prevention. Lancet.

[64] Burci GL, Moon S, Viñuales J. A Global Pandemic Treaty Should Aim for Deep Prevention. Graduate Institute; 2021. Available from: https://www.graduateinstitute.ch/communications/news/global-pandemic-treaty-should-aim-deep-prevention. Accessed May 15, 2023. 10.1016/S0140-6736(21)00948-X 33932327

[65] Chappell B. The ‘Time Has Come’ For A Global Pandemic Treaty, WHO’s Tedros Says. National Public Radio; 2021. https://www.npr.org/sections/coronavirus-live-updates/2021/05/31/1001943709/the-time-has-come-for-a-global-pandemic-treaty-whos-tedros-says. Accessed May 15, 2023.

[66] Ellis R. World Leaders Call for International. Pandemic Treaty. WebMD; 2021. Available from: https://www.webmd.com/lung/news/20210331/world-leaders-call-for-international-pandemic-treaty. Accessed March 31, 2023.

[67] The Conversation. The WHO’s International Pandemic Treaty: Meaningful Public Engagement Must Inform Canada’s Negotiations. The Conversation; 2023. Available from: https://theconversation.com/the-whos-international-pandemic-treaty-meaningful-public-engagement-must-inform-canadas-negotiations-203747. Accessed March 31, 2023.

[68] Alcázar L, Bhattacharya D, Charvet E, Kida T, Mushi D, Ordóñez A, te Velde DW. COVID-19 in the Global South: Impacts and Policy Responses. Southern Voice Occasional Paper Series, 69. 2021. Available from: https://southernvoice.org/wp-content/uploads/2021/02/COVID-19-Impacts-Policy-Responses-Alcazar-et-al-2021.pdf. Accessed April 24, 2023.

[69] Winchester N. COVID-19 Vaccinations: Is the Global South Falling Behind? House of Lords Library; 2021. Available from: https://lordslibrary.parliament.uk/covid-19-vaccinations-is-the-global-south-falling-behind/. Accessed Accessed April 20, 2023.

[70] Gostin LO, Klock KA, Ginsbach K, Halabi S, Hall-Debnam T, Lewis J, et al. Advancing Equity in the Pandemic Treaty. Georgetown Law Faculty Publications and Other Works. 2513. 2023. Available from: https://ssrn.com/abstract = 4473649. Accessed June 28, 2023.

